# Obesity, body fat distribution and eye diseases

**DOI:** 10.1007/s40519-024-01662-8

**Published:** 2024-05-06

**Authors:** Francesca Bosello, Angiola Vanzo, Chiara Zaffalon, Luca Polinelli, Filippo Saggin, Erika Bonacci, Emilio Pedrotti, Giorgio Marchini, Ottavio Bosello

**Affiliations:** 1 Department of Surgery, Dentistry, Maternity and Infant, Ophthalmology Clinic, University of Verona, Verona, Italy; 2 Food Hygiene and Nutrition Unit, Azienda ULSS 8, Berica, Veneto, Italy; 3 Department of Engineering for Innovation Medicine, Ophthalmology Clinic, University of Verona, Verona, Italy; 4 Department of Medicine, University of Verona, Verona, Italy

**Keywords:** Obesity, Visceral obesity, OSAS, Eye disease, Cataract, Glaucoma, Papilledema, AMD, Diabetic retinopathy, Uveitis, Dry eye syndrome

## Abstract

**Background:**

The prevalence of obesity, a chronic disease, is increasing, and obesity is now considered a global epidemic. Eye diseases are also increasing worldwide and have serious repercussions on quality of life as well as increasingly high costs for the community. The relationships between obesity and ocular pathologies are not yet well clarified and are not pathologically homogeneous: they seem to be somehow linked to excess body fat, especially to the distribution of adipose tissue and its ectopic deposits.

**Purpose:**

Our objective was to examine the associations between obesity and anthropometric indices, including body mass index (BMI), waist circumference (WC), and the waist/hip ratio (WHR), and the risk of most widespread eye diseases, with particular attention given to the most significant metabolic mechanisms.

**Methods:**

This article provides a narrative overview of the effect of obesity and anthropometric measurements of body fat on prevalent eye diseases. We used the MEDLINE/PubMed, CINAHL, EMBASE, and Cochrane Library databases from 1984 to 2024. In addition, we hand-searched references from the retrieved articles and explored a number of related websites. A total of 153 publications were considered.

**Results:**

There is significant evidence that obesity is associated with several eye diseases. Waist circumference (WC) and the waist/hip ratio (WHR) have been observed to have stronger positive associations with eye diseases than BMI.

**Conclusions:**

Obesity must be considered a significant risk factor for eye diseases; hence, a multidisciplinary and multidimensional approach to treating obesity, which also affects ocular health, is important. In the prevention and treatment of eye diseases related to obesity, lifestyle factors, especially diet and physical activity, as well as weight changes, both weight loss and weight gain, should not be overlooked.

**Level of evidence:**

Level V narrative review.

## Introduction

Obesity represents a public health challenge worldwide. Moreover, eye diseases are serious worldwide health problems that negatively impact quality of life and increase healthcare costs.

The relationships between obesity and eye diseases are not yet well clarified and are not pathologically homogeneous; therefore, there is a need to investigate them further, particularly to evaluate the risk factors linked to the lifestyles of the populations examined and the associations with different anthropometric measurements of obesity.

This article provides a narrative overview of the effect of obesity and anthropometric measures of body fat on prevalent eye diseases. We used the MEDLINE/PubMed, CINAHL, EMBASE, and Cochrane Library databases from 1984 to 2024. In addition, we hand-searched references from the retrieved articles and explored a number of related websites. A total of 153 publications were considered.

Our aim was to examine associations between several anthropometric measures of obesity, including body mass index (BMI), waist circumference (WC), and the waist/hip ratio (WHR), and the risk of more prevalent eye diseases, with particular attention given to the most significant metabolic mechanisms.

## Notes on obesity

**Fig. 1:**
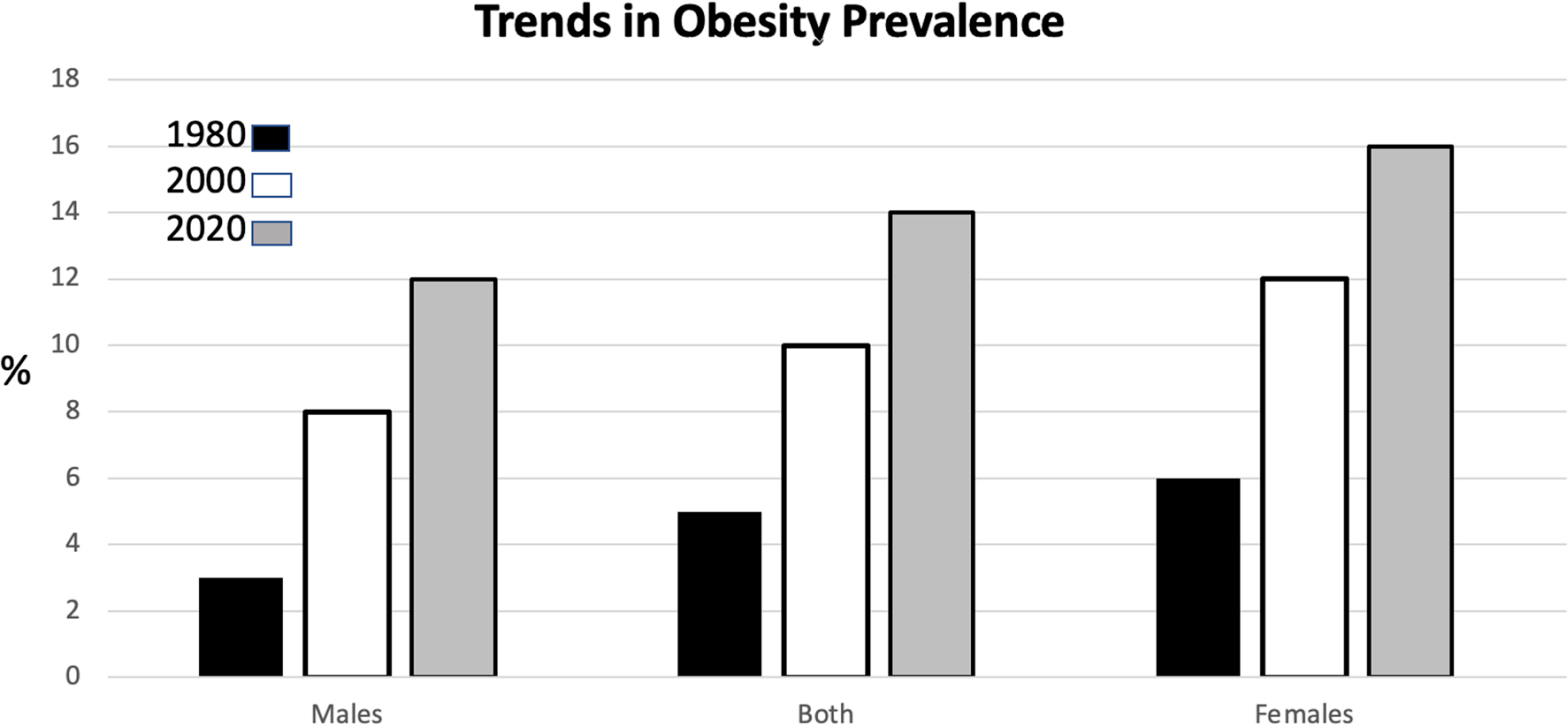
Global trend. Age-standardized global prevalence of obesity in men and women over 20 years of age per year (1980–2019), modified [1, 2]. (https://ghdx.healthdata.org/record/global-burden-disease-study-2019-gbd-2019-covariates-1980-2019)

The Global Burden of Disease Group (Institute for Health Metrics and Evaluation, Global Burden of Disease Study 2019, GBD 2019) reported that the prevalence of obesity worldwide has approximately tripled since 1980 in more than 70 countries and has increased steadily in most other countries, now exceeding two billion people worldwide (Fig. 1) [1, 2].

Worldwide, at least 2.8 million people die each year due to overweight- or obesity-related pathologies, and it is estimated that 35.8 million (2.3%) of the global disability-adjusted life years (DALYs) are caused by overweight or obesity.

Increased BMI is an important risk factor for noncommunicable diseases, such as the following:

1. cardiovascular diseases (mainly heart disease and stroke), which have been the leading cause of death in recent decades;
2. type 2 diabetes;
3. musculoskeletal disorders (in particular osteoarthritis, a highly disabling degenerative joint disease);
4. certain cancers (including endometrial, breast, ovarian, prostate, liver, gallbladder, kidney and colon);
5. eye diseases.

The risk of these noncommunicable diseases increases as BMI increases.

Several studies have shown that insulin resistance is a predictor of disease risk that is sometimes stronger than BMI at any BMI. According to a prospective follow-up of 5.5 years, incidence of prediabetes/type 2 diabetes was lower among insulin-sensitive obese subjects than among insulin-resistant obese subjects (31.3% vs 48.7%) and was greater among insulin-resistant nonobese subjects than among insulin-sensitive nonobese subjects (47.1% vs. 26.0%) (*P* = 0.0024). Insulin-sensitive obese subjects had a 40% lower incidence of diabetes than insulin-resistant obese subjects. Moreover, nonobese people with insulin resistance have an 80% greater risk of developing diabetes than insulin-sensitive individuals [3].

Genetics is a major point of interest in research into the precise mechanisms by which individuals with excessively high BMIs progress on the path to insulin resistance, dyslipidemia, or hypertension [4].

A sedentary lifestyle and a decrease in overall physical activity in combination with the consumption of unhealthy diets (high in sugar and refined carbohydrates) associated with a myriad of genetic, endocrine, metabolic and environmental factors are currently considered the main common causes of the obesity epidemic that has taken over in many parts of the world in recent decades.

## Obesity and eye diseases

Like obesity, eye diseases are serious public health problems. In 2015, Bourne et al. reported that mild visual impairment affected 118.5 million people, moderate to severe visual impairment affected 216.6 million people, and blindness affected 36.0 million people worldwide [5]. The prevalence of age-related eye diseases is expected to increase worldwide as the population ages [5]. According to the Global Burden of Disease Study (2021), 43.3 million people were estimated to be blind in 2020, 33.6 of whom were aged 50 and older; the number of blind people of all ages is expected to increase to 61.0 million by 2050 [6]. Moreover, the number of people with moderate to severe visual impairments is expected to increase worldwide from 216.6 million in 2015 to 587.6 million by 2050 [5]. In 2021, Steinmetz et al. published estimated data on global causes of visual impairment, including uncorrected refractive defects, cataracts and other age-related ocular pathologies [7].

Although the negative effects of obesity on health have been and are still the subject of multiple studies, the effects of excess weight on eye health are still poorly understood. In fact, obesity is a health problem that affects the whole body and has significant repercussions on the visual system. There are many eye diseases associated with obesity (Table 1); among the most studied are glaucoma, cataracts, age-related macular degeneration (AMD), diabetic retinopathy (DR), and dry eye syndrome (DES) [8, 9].

**Table 1:** Ocular disorders associated with obesity (da, Kapoor et al. 2022, modified) [9]

Ocular disorders associated with obesity
Cataract—posterior subcapsular and cortical cataracts
Glaucomatous optic neuropathy
Age-related macular degeneration
Retinal artery and vein occlusion
Papilledema
Uveitis
Dry eye syndrome
Ocular diseases associated with obstructive sleep apnea:
Floppy eyelid syndrome
Central serous chorioretinopathy
Normal tension glaucoma
Nonarteritic anterior ischemic optic neuropathy
Myopia, astigmatism, amblyopia, strabismus and exotropia—associated with syndromic obesity

The effects of obesity on the ophthalmic system are complex and varied. Regarding the etiopathogenetic mechanisms that can link obesity to eye diseases, inflammation, oxidative stress and hormonal imbalances are certainly important [10]. Metabolic syndrome and related cardiovascular disorders may also be involved in eye disease pathogenesis [10].

In a systematic review of prospective population-based studies, Ling et al. reported high-quality evidence-based data on the associations between obesity, with different anthropometric measures (BMI, WC, WHR), and four main age-related ocular pathologies: cataracts, glaucoma, AMD and diabetic retinopathy [11]. Interestingly, there was different evidence due to different populations, mainly Western or Asian populations, or due to the different anthropometric measures reported in the studies. For example, significant correlations between obesity, defined by BMI, and elevated intraocular pressure (IOP) have emerged in Asian populations. Obesity defined by high BMI was positively associated with AMD in Western populations but not in Asian populations [11]. Again, obesity was positively associated with DR in Western populations, while an inverse association was found in Asian populations [11]. We will report these data in more detail in the next paragraphs. In general, there is evidence that WC is generally a better indicator than BMI for the risk of both AMD and DR in Western, Asian, and other non-Western populations [11].

In Asian and other non-Western populations, a high BMI could have a protective effect on eye diseases [11]. Could this be a case of an obesity paradox [12, 13]?

To clarify these conflicting observations on the involvement of obesity and visceral fat in ocular diseases, more longitudinal studies are needed, particularly on abdominal adiposity and the effectiveness of weight loss in preventing or improving age-related eye diseases.

### Obesity and cataracts

Lens opacities, specifically cataracts, are usually age-dependent ocular pathologies [9, 14]. Some population surveys have revealed significant associations between obesity and cataracts. In 1995, Glynn et al. found a strong correlation between BMI and incident cataracts, particularly posterior subcapsular cataract (PSC) and nuclear cataract; considering BMI as a continuous variable, a 2-unit increase in BMI value predicted a 12% major risk of cataract [14].

In 2000, Schaumberg et al. analyzed data from the Physicians' Health Study and reported that among 17,150 males followed up for 5 years, BMI and WHR were found to be independent risk factors for incident cataracts [15]. Glynn et al. in 2009, reported that the type of cataract that is most strongly associated with high BMI is cortical cataract [16].

There is evidence that, compared to obesity assessed by BMI, abdominal obesity is a better indicator for any type of incident cataract. In fact, waist circumference (WC), waist-to-hip circumference (WHR) and waist-to-height ratio (WHtR) are more reliable and specific indicators of intra-abdominal fat and visceral obesity than BMI is [17]. Metabolic syndrome and its variations appear to predict the 5-year incidence of cortical cataracts and PSC. Information on metabolic syndrome, collected at each follow-up visit, accurately predicts the risk of different cataract subtypes in older people [18].

Regarding cataract pathogenesis, Schaumberg et al., analyzing data from the Physicians' Health Study, reported that elevated levels of CRP, a marker of systemic inflammation, in 834 healthy males followed up for 11 years are associated with an increased risk of incident cataracts [19]. In 1984, it was hypothesized and demonstrated in rats that increased levels of oxidative stress cause oxidative damage to the lens [20]. Moreover, obesity may correlate with subclinical systemic inflammation and oxidative stress, which in turn promote the development of cataracts [21]. There are also well-established systemic risk factors for adult-onset cataracts, which are frequently associated with obesity, such as hypertension [22], hyperlipidaemia [23], diabetes, and insulin resistance [18, 24].

On the other hand, several studies have revealed that a higher level of physical activity is associated with a reduced incidence of cataracts due to reduced levels of oxidative damage [25, 26].

Cohort studies on those who practiced physical activity, such as walking or running, have shown data compatible with a lower risk of cataract onset in adulthood [27].

In conclusion, there is evidence from the literature that obesity, defined by BMI, is associated with an increased risk of age-related cataracts and posterior subcapsular and cortical cataracts in adults [28].

### Obesity and glaucomatous optic neuropathy

Glaucoma is a multifactorial eye disease and is the primary cause of irreversible blindness worldwide. The most common type of glaucoma, primary open-angle glaucoma (POAG), affects approximately 80–100 million people worldwide [29]. Neurodegeneration, neuroinflammation and progressive loss of retinal ganglion cells (RGCs) and their axons constitute the main pathophysiological aspects of optic neuropathy [29, 30].

At an early stage, clinical symptoms are usually silent, so visual function can often be irreversibly impaired at diagnosis. It is therefore crucial to determine and evaluate the risk factors for the development of glaucoma to propose useful methodologies to promote early diagnosis and, consequently, interventions aimed at detecting the progression of the disease [31]. Elevated intraocular pressure (IOP) is the first and most frequently called upon risk factor in primary open-angle glaucoma [32, 33]. Kim et al. investigated the 5-year progression of glaucoma and reported that baseline age is a significant risk factor for progression from POAG suspect to definite POAG [33]. Other significant risk factors for progression to POAG are high baseline IOP, high BMI, high level of education, and high hematocrit level [33]. Multiple factors, such as genetic burden [34], environmental factors [35], and systemic diseases [36], are involved.

Open-angle glaucoma, both hypertensive [37] and normotensive [38] glaucoma, is significantly associated with obstructive sleep apnea syndrome (OSAS). In these patients, glaucomatous damage may occur in the hypertensive form due to increased intraocular pressure induced by overstimulation of the sympathetic system [37] or in the normotensive form due to insufficient vessel perfusion at the level of the peripapillary plexus caused by hypoxia [38]. Refer to the paragraph on “Obesity, OSAS and eye diseases”. Yamada et al. evaluated glaucoma progression in relation to the presence of OSAS. They observed that the speed of visual field deterioration was greater in open-angle glaucoma patients with OSAS than in patients without OSAS. They measured the total plasma oxidant capacity and found that glaucomatous patients with OSAS had greater plasma oxidative capacity and faster glaucoma progression than those without OSAS [39].

One of the most controversial aspects is whether obesity represents a risk factor for POAG or whether, in certain circumstances, it could even be a protective factor [40]; in this sense, the contributions of the literature still seem to be unconvincing and are the object of discussion.

Numerous investigations have confirmed this association, reporting a significant relationship between obesity and the incidence of glaucoma.

Wise et al., in a prospective cohort study of African-American women, demonstrated that POAG was significantly associated with BMI, waist circumference, waist-to-hip ratio, and both long-duration and high-intensity current smoking [41]. Other studies have also reported associations between BMI and abdominal obesity, elevated IOP and glaucoma [42–44]. A population-based survey in the United States confirmed that obesity (BMI > 30) is significantly related to the prevalence of glaucoma [45]. To further confirm this finding, a prospective study in Korea revealed that POAG occurred more frequently in subjects with a BMI > 30 than in those with a BMI between 18.5 and 22.9 [46].

Lin et al. used “two-sample multivariate Mendelian randomization” for the first time and reported that BMI and hip circumference, but not waist circumference, are able to increase the risk of POAG [47]. In contrast, in a population study in central India, Nangia et al., using a multivariable analysis, found that an increased prevalence of glaucoma was significantly correlated with a lower body mass index (*P* = 0.01) [48]. These conflicting observations may depend on the different ethnicities of the subjects studied and other possible confounding factors, such as lower blood concentrations of hemoglobin and lower levels of education [48].

Pasquale et al., analyzing data from two American population studies (Nurses Health Study and Health Professionals Follow-up Study), reported an interesting inverse relationship between BMI and normal tension glaucoma in women but not in men. Considering the different distributions of weight, waist and hip circumference were evaluated, and the data showed that people with the largest hip circumference had a 27% lower risk of POAG than people with the smallest hip circumference [43]. These data reflect different pathogenetic aspects of glaucoma and the importance of evaluating different anthropometric measures together with BMI.

From the analysis of the results from the modest specific literature, it seems to be possible to affirm that BMI is generally associated with an increased risk of POAG.

Some hypotheses have been proposed about the mechanisms by which obesity may increase the risk of POAG. Zarei et al. reported that the increase in androgens present in obese individuals is responsible for the greater level of oxidative stress in obese individuals than in normal-weight individuals. This, in turn, increases the level of oxidative damage in the aqueous humor outflow system at the trabecular meshwork, thus blocking the aqueous humor outflow channel and significantly increasing the intraocular pressure and the risk of POAG [49].

Pezzino et al. suggested that in obesity, there is a condition of intestinal dysbiosis with systemic diffusion of proinflammatory metabolites and bacteria; this could induce systemic inflammation through intestinal permeability, in which TLR4 activation plays a major role and could alter retinal and ocular barriers. This event, in turn, could contribute to local inflammation (neuroinflammation), leading to RGC death and neurodegeneration [50].

In conclusion, evidence from the literature is still scarce but suggestive of significant associations between BMI, waist circumference and hip circumference and the risk of glaucoma. These observations can be considered very useful for glaucoma prevention and treatment via a more systemic approach to this ocular pathology.

### Obesity and papilledema

Papilledema is a condition characterized by edema of the optic nerve head secondary to increased intracranial pressure (ICP). This condition differs from other causes of optic disc edema because visual function in the acute phase is usually normal [51].

Papilledema is caused by the transmission of elevated ICP to the subarachnoid space surrounding the optic nerve, which hinders axoplasmic transport within ganglion cell axons.

The most common cause of papilledema, especially in obese patients younger than 50 years of age, is idiopathic intracranial hypertension (IIH).

In recent years, some studies have shown that obesity, particularly BMI and body fat percentage, may also be associated with ICP dynamics, with a direct correlation between elevated BMI and increased ICP [51–54].

Furthermore, a 2022 study by Westgate et al. demonstrated for the first time in rodents that diet-induced obesity leads to an increase in ICP and the consequent development of clinically relevant symptoms [55].

IIH is a metabolic disease characterized by an increase in ICP with no identifiable cause, apart from obesity being the main risk factor. The condition mainly affects women, and 90% of patients affected are obese [56].

IIH has significant morbidity, and the most common symptoms are chronic headache, cognitive impairment, and visual disturbances due to papilledema [57, 58].

Increased BMI in IIH patients is associated with an increased grade of IIH. The relationship between BMI and visual outcomes in IIH patients was fairly clarified in a retrospective review of 414 consecutive IIH patients between 1989 and 2010, of whom 158 had a BMI ≥ 40 (Obese Class III WHO) and 172 had a BMI between 30 and 39.9 [58]. A higher BMI at diagnosis is associated with an increased risk of severe visual loss in patients with IIH, independent of other known risk factors. Patients with idiopathic intracranial hypertension (IIH) and a BMI ≥ 40 years were more likely to have severe papilledema at the first neuroophthalmological examination than were those with a lower BMI (*P* = 0.02). There was a trend toward more severe visual loss in one or both eyes. Logistic regression models revealed that a 10-unit increase in BMI increased the odds of severe visual loss by 1.4 times after equalizing for sex, race, high blood pressure, and OSAS [58].

The association of a more severe tendency toward papilledema and vision loss associated with increased BMI suggests that very obese IIH patients should be closely monitored for the progression of visual field loss. Wall et al. demonstrated the beneficial effect of acetazolamide tablets combined with a low-sodium weight reduction diet on visual function [59]. If progression is observed, early bariatric surgery may be especially important for IIH patients with multiple risk factors for severe vision loss, including black race, male sex, anemia, hypertension, and very high BMI [52].

Although aggressive weight loss efforts (by bariatric surgery) often lead to remission of the disease, it remains to be determined whether these efforts also reduce the risk of severe visual loss in very obese IIH patients.

To assess the correlation between weight reduction and visual outcome in overweight patients with IIH, Koc Feray et al. retrospectively studied 39 newly diagnosed overweight patients (BMI > 25) with IIH [60]. Weight reduction achieved with medical treatment has been associated with significant improvements in visual acuity, visual field and papilledema. Adherence to an appropriate treatment program should be encouraged in overweight patients with IIH.

In conclusion, the severity of papilledema and visual loss are associated with increased BMI in individuals with IIH [61]. Female sex is clearly more strongly affected by IIH, and the necessity of a weight loss program involving diet or bariatric surgery in severely obese patients has been well established to avoid visual function loss and other complications [56].

### Obesity and age-related macular degeneration (AMD)

Age-related macular degeneration (AMD) is a typical disease of old age and is the most common cause of blindness in developed countries [62]. AMD is characterized by degeneration of the photoreceptors of the macula, which causes significant central visual deficit. AMD is a complex, multifactorial disease for which numerous modifiable and nonmodifiable risk factors have been identified. The most proven consistent risk factor for developing advanced AMD is smoking [63].

There is some evidence that, in Western populations, obesity is associated with AMD but not with its progression. The exact mechanisms underlying the association between obesity and AMD risk are unclear. It has been reported that the WHR is a potentially better marker for AMD than BMI is [64].

Public health initiatives to reduce the risk of AMD show promise in weight control and physical activity. A longitudinal study investigating the effect of weight loss on the incidence of AMD revealed that a ≥ 3% reduction in the WHR was associated with a decrease in the incidence of AMD [65]. A meta-analysis confirmed that physical activity is protective against the development of both early and late AMD, underscoring the importance of remaining active throughout life [66].

Multiple studies have confirmed that obesity is characterized by an energy-dense diet as well as a high-fat diet [67]. Agron et al. reported that high dietary fat intake is associated with a greater risk of AMD progression [68].

Pameijer et al. also reported that a high consumption of specific nutrients, especially antioxidant vitamins and omega 3 fatty acids, is associated with reduced progression of late AMD [69]. There is also epidemiological evidence that antioxidant supplements and adherence to a Mediterranean-type diet, with high consumption of vegetables and whole grains and low consumption of red meat, are associated with a reduced risk of both early and late AMD progression [70]. High alcohol consumption is instead associated with a greater risk of developing AMD [71].

In conclusion, smoking, nutrition and physical activity are important factors involved in the development of AMD. In particular, physical activity is associated with a lower risk of AMD in many populations, with important public health implications. The possibility of carrying out even modest physical activity is already protective against the onset of AMD. Further studies are needed to confirm the protective effect of physical activity on the development and/or progression of AMD, as well as adequate guidelines regarding the type and amount of physical activity that are truly effective [66].

### Obesity and diabetic retinopathy

Obesity and diabetes are positively associated with the development of diabetic retinopathy (DR), its progression and its complications among older adults in the US [72–74]. Yau et al. examined the global prevalence and major risk factors for diabetic retinopathy (DR) and vision-threatening diabetic retinopathy (VTDR) among people with diabetes in a systematic literature review of 35 studies: 34.6% of diabetic patients were affected by any DR, 6.96% by proliferative diabetic retinopathy (PDR), 6.81% by diabetic macular edema, and 10% by VTDR [75]. In a Chinese community-based cross-sectional study, 40% of diabetic patients were affected by diabetic retinopathy. They were more likely to be elderly (*P* = 0.0003), male (*P* = 0.018), and hypertensive (*P* < 0.0001) and to have a high body mass index (*P* < 0.0001), metabolic abnormalities, and a longer duration of diabetes (*P* < 0.0001) [76]. In a 2024 prospective cohort study, Chen et al. reported that visceral obesity, measured by the visceral adiposity index (VAI), lipid accumulation product (LAP) and Chinese VAI (CVAI), is independently associated with an increased risk of new-onset DR in Chinese patients with type 2 diabetes [77]. These findings may suggest the need to incorporate monitoring regulated indices of visceral obesity in routine clinical practice to improve the prevention of DR.

Hyperleptinemia present in both obese and diabetic patients not only promotes oxidative stress, but also increases ocular vascular endothelial growth factor [78], constituting a pathogenetic cofactor of neovascularization in proliferative DR [79–81]. Hypertension and hyperlipidemia [82], which are very often associated with obesity, can also cause endothelial dysfunction [83, 84].

In Asian populations, particularly those of South and Southeast Asia, obesity appears to exert a protective effect on the incidence of DR in diabetic patients [85]. This inverse relationship constitutes the so-called "obesity paradox", in which a higher BMI seems to confer a protective advantage over a low or normal BMI [13]. Gupta et al. hypothesized that thinner patients could have more severe diabetes than their obese counterparts and therefore would be at greater risk of DR [83]. According to these authors, a longer duration of diabetes is generally associated with reduced insulin secretion capacity, which can lead to a reduction in BMI [83]. Another important consideration is that in South-Southeast Asian populations, the anatomical body conformation is different from that of Western populations, and the clinical use of adipose localization indicators such as the WHR and WC has become increasingly common. In fact, there are studies indicating that visceral obesity, as measured by the visceral adiposity index, is independently associated with an increased risk for new-onset DR in Chinese patients with diabetes [77]. Furthermore, the WHR is a more clinically relevant risk marker than BMI for individuals with type 2 diabetes [86].

Finally, there are data that support a protective effect of physical activity against DR, with greater evidence in the most severe DR with initial visual deficit [87].

### Obesity and retinal vessel changes

The retinal vascular caliber is a surrogate marker of microvascular disease and a predictor of cardiovascular events.

Retinal microvascular changes can occur in individuals with cardiovascular involvement associated with obesity. In particular, associations emerge between ten-year trajectories of high BMI and WHtR (waist–height ratio) and narrow retinal arteriolar caliber in children and are clearly evident in middle age; venular caliber showed a late association with child WHtR but not with BMI [88]. Over the course of life, adiposity therefore appears to exert its first negative effects more and earlier on arterial retinal microcirculation [88].

Another longitudinal study on young and adult obese subjects revealed a narrower arteriolar caliber and wider venular caliber, independent of conventional cardiovascular risk factors [89].

In childhood obesity, there is evidence of proinflammatory and pro-oxidative processes. The excessive accumulation of adipose tissue triggers inflammation of adipose tissue and the release of a number of bioactive substances [90, 91]. The bioavailability of nitric oxide (NO) has been shown to decrease in individuals with obesity. NO is a key endothelial-derived relaxing and vasodilator factor that regulates perfusion. Low levels of NO in childhood obesity may be a contributing factor to narrower arteriolar diameters [91].

To the same extent, there is evidence that markers of inflammation are associated with larger retinal venules. Inflammation may therefore be one of the main mechanisms through which obesity alters retinal microcirculation [92].

The results of a cross-sectional study of 1555 Chinese adults aged ≥ 50 years with no history of ocular disease showed that BMI and waist-to-hip ratio are positively associated with increased macular vessel density (VD) on optical coherence tomography angiography, both in the superficial capillary plexus and deep capillary plexus. The different manifestations of retinal microvascularization may therefore reflect the distinct effects of body composition on macular vessel alterations and disease onset [93].

Obesity has also been recognized as a significant risk factor for retinal vein occlusion and arteriolar emboli in other studies, with a significant tendency to increase risk across all quartiles of BMI [94–96].

It has been documented by studies from the National Health and Nutrition Examination Survey, 1999–2006, that a high BMI is related to an increased risk of developing both diabetes mellitus (DM) and retinal vein occlusion (RVO) [97].

Several reviews have shown evidence supporting the association of hypercoagulability disorders with obesity and metabolic syndrome. It is also known that retinal arterial and venous occlusions are associated with hypercoagulability or hyperviscosity syndromes. This provides further support for a reliable association between obesity and retinal vascular occlusive disease [98–100].

Retinal vascular occlusions, particularly arterial occlusions, can also be caused by hypoxia in OSAS, which causes severe dysfunction in the self-regulation of blood vessels, leading to hypertensive retinopathy in the early phase and vessel retinal occlusions later [89].

Recently, low folate levels have been observed in many patients with retinal vascular diseases, such as retinal vascular occlusions, diabetic retinopathy, and age-related degeneration. It has been suggested that folic acid supplementation might have a protective effect in patients with retinal vascular diseases [101].

A Korean population‐based study revealed evidence that obesity has a different effect on the incidence of RVO in the presence and absence of DM. In people with DM, a lower BMI was associated with an increased risk of RVO, and a higher BMI was associated with a lower risk of RVO. In people without DM, the correlation was reversed: a lower BMI was associated with a lower risk for RVO, and vice versa. This inverse association is related to the degree and duration of DM [102]. Although this study concluded that a high BMI in patients with DM is associated with a reduced risk of developing RVO, care should be taken not to infer that a higher BMI is always desirable. In fact, in the Korean study, all subjects had a significantly greater risk of RVO than did those in the normal weight group without DM. In addition, as the severity and duration of DM increase, the incidence of RVO also increases. The inverse association between BMI and RVO incidence was therefore found only in subjects with DM.

These observations seem to constitute a kind of "obesity paradox", a concept that has already been discussed and widely contested [103]. The main criticism comes from considering BMI as the only indicator of obesity, while it is now known that the distribution of body fat is crucial [104–106].

### Obesity and uveitis

The term uveitis refers to an inflammatory process affecting the uvea. From an etiopathogenetic point of view, uveitis can be divided into infectious, noninfectious or masquerade, and these pathologies represent a major cause of blindness [107]. The literature is practically orphaned of contributions on obesity and uveitis. Considering that obesity is a chronic disease characterized by an increase in adipose tissue and a corresponding increase in the production of inflammatory cytokines and an overall systemic subclinical state of inflammation, it is possible to think of a link, albeit indirect, with uveitis [108].

A mouse model of human autoimmune uveitis, experimental autoimmune uveitis (EAU), is used to better understand the immunobiology of uveitis. A study of EAU in a high-fat diet (HFD)-induced obesity model revealed that the severity of EAU was significantly greater in wild-type mice than in mice with melanocortin 5-induced HFD deficiency [109]. It has also been shown that as EAU progresses, obese mice lack the regulatory immunity that provides protection from EAU [109]. This report demonstrated that obesity exacerbates autoimmune uveitis and inhibits post-EAU regulatory immunity via the melanocortin 5 receptor [109]. It is therefore suggestive to believe that obesity may contribute to the exacerbation and severity of autoimmune uveitis.

Behçet's disease (BD) is a chronic multisystem autoinflammatory vasculitis characterized by, among other manifestations, uveitis. Some studies have shown that patients with BD are more likely to have metabolic syndrome (MetS), which is reported to be present in 25.52% of patients [110]. Consistent with previous reports, a study by Chen et al. confirmed an important association between BD and MetS: the risk of metabolic syndrome was 126% greater in the BD group than in the healthy control group [111]. These findings revealed that BD is significantly associated with insulin resistance, diabetes mellitus, and hypertension [111].

Considering that abdominal adiposity is one of the fundamental indicators of metabolic syndrome, an indirect link may exist between obesity, especially visceral-abdominal obesity, and uveitis. However, further studies on obesity and uveitis are needed.

### Obesity and dry eye syndrome

Dry eye syndrome is a very common eye disorder that often significantly affects quality of life and may be linked to systemic inflammatory diseases, localized eye diseases or frequently used medications. The main descriptive definition of dry eye (DEWS II, 2017) is a multifactorial disease of the ocular surface characterized by tear film dysfunction caused by tear production deficiency or excessive evaporation, accompanied by ocular symptoms such as ocular discomfort, pain, itching, light sensitivity, fatigue or strain, heavy or foreign body sensation, and discharge [112].

Recent studies evaluating its etiopathogenesis associated a high incidence of dry eye with body fat composition and lipid metabolic changes [113]. Ho et al. reported an association between adiposity measured by body fat percentage and symptoms of dry eye in the general adult population [114]. The study demonstrated a moderate positive correlation between body fat percentage and symptoms of dry eye: women with body fat values of 30% and above and men with body fat values of 20% and above were more likely to report dry eye on the SFDEQ (Short Form Dry Eye Questionnaire) [114].

With increasing life expectancy and increasing age, people tend to have a sedentary lifestyle [115]. This lifestyle is one of the main causes of obesity and associated diseases and/or complications, including hypertension, diabetes, metabolic syndrome and highly prevalent ones. The source of low-grade inflammation in obesity, adipose tissue, is rich in proinflammatory macrophages that produce proinflammatory markers. These markers exert some negative impacts on tear production, thus linking pathogenically obesity and dry eye disease [113].

Therefore, lifestyle intervention may be a promising management option for dry eye disease. Ismail et al. evaluated the effect of lifestyle modification as a complementary treatment for DES in a randomized, controlled trial of sixty obese hypertensive adults with DES. The authors observed that dry eye obese patients who maintained a physical activity program for 6 months, characterized by thirty minutes of intense aerobic exercise, 3 times a week, alone or combined with a Mediterranean diet, revealed significant improvements in dry eye symptoms, in both subjective and objective tests, which were better in the group following the low-calorie Mediterranean diet [116].

In conclusion, an albeit moderate but positive correlation was found between body fat percentage and dry eye symptoms. There is also evidence that weight loss, caloric restriction and an active lifestyle are associated with significant improvements in patients with dry eye.

## Eye disorders associated with obesity and obstructive sleep apnea syndrome (OSAS)

Obstructive sleep apnea syndrome (OSAS) is a medical condition characterized by interruptions in breathing during sleep due to total or partial obstruction of the upper airway.

OSAS occurs in the general population in approximately 3–7% of men and 2–5% of women [117]. Important clinical symptoms of OSAS include snoring, daytime sleepiness, restless sleep, morning fatigue, and headache.

### Obesity and OSAS often coexist

Similar to obesity, obstructive sleep apnea negatively affects multiple organs and systems, with particular relevance to the cardiovascular system [118]. Several other conditions associated with OSAS, such as arterial hypertension, insulin resistance, systemic inflammation, visceral fat accumulation and dyslipidemia, are also more frequently associated with obesity. There is also evidence that subjects with OSAS are characterized by a greater degree of insulin resistance than BMI-matched controls [119].

Obesity is a common finding and an important pathogenetic factor in OSAS, both in adults [120, 121] and children [122–124]. Jehan et al. reported that there is a linear correlation between obesity and OSAS [117]. Most adult patients with OSAS are characterized by central obesity and increased visceral fat [125], the latter of which is associated with neck adiposity, increased upper airway fat [126], and metabolic abnormalities [127] even in normal-weight individuals. Visceral fat expansion increases the risk of developing insulin resistance, type 2 diabetes, atherosclerosis, OSAS, steatohepatitis, and cardiovascular and cerebrovascular disease [128].

Sex-related differences in the amount of visceral fat could contribute to the greater prevalence of OSAS in males [129]. It is conceivable that OSAS and obesity may interact and potentiate their harmful consequences [130, 131].

**Fig. 2:**
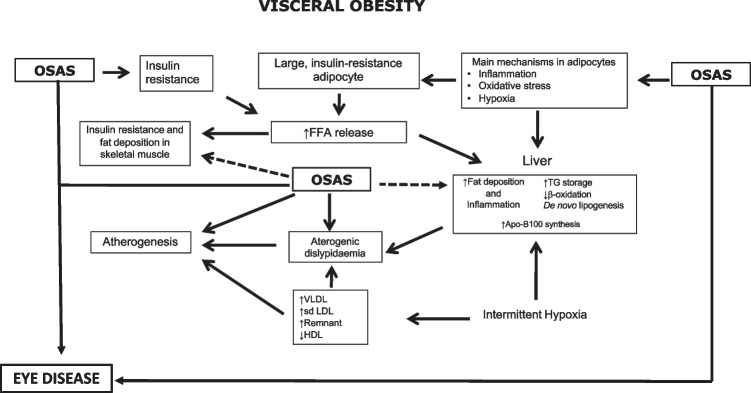
Schematic representation of the metabolic consequences of visceral obesity and OSAS on adipocytes, skeletal muscle, liver and vascular wall and their impact on ocular pathologies. TG: triglyceride; FFA: free fatty acid; Apo-B100: apoprotein B100; VLDL: very low-density lipoprotein; sdLDL: small, dense lipoprotein; HDL: high-density lipoprotein (Bonsignore et al., 2012, modified) [136]

Vgontzas et al. reported that OSAS patients had significantly more visceral fat than obese controls (< 0.05), and indices of sleep breathing disturbances were positively correlated with visceral fat but not with BMI or total or subcutaneous fat [132], suggesting that there is a strong independent association between OSAS, visceral obesity and insulin resistance [132, 133]. Increased visceral fat and inflammation of adipose tissue have recently been found in morbidly obese insulin-resistant subjects compared to insulin-sensitive subjects of equal weight [134, 135]. Bonsignore et al. [136] hypothesized that the underlying cause is peculiar metabolic dysfunction of visceral adipocytes, which is well represented in Fig. 2.

In the general picture of common pathogenetic mechanisms shared by OSAS and obesity, there is also an emerging role for oxidative stress, which is linked to low-grade inflammation and increased sympathetic activity due to hypoxia [130, 136–140].

OSAS obese patients benefit significantly from weight reduction; if obesity is high-grade, bariatric surgery may also be considered [141].

### OSAS and eye diseases

OSAS also has repercussions on eye health. It has been demonstrated that some ocular pathologies are frequently associated with OSAS both in adults and in children [142, 143]: the eyes can even represent the first alarm bell for recognizing, diagnosing and intervening early in obstructive sleep apnea syndrome [9].

A recent systematic review and meta-analysis revealed significant associations between OSAS and many ocular pathologies. Those that seem to have the closest pathophysiological links are non-arteritic anterior ischemic optic neuropathy (NAION), proliferative diabetic retinopathy (RDP), central serous chorioretinopathy (CSCR) and floppy eyelid syndrome (FES) [9, 144]. Other ocular pathologies, such as vascular occlusions, papilledema, and glaucoma, that may be linked to OSAS are discussed in the previous paragraphs.

Nonarteritic anterior ischemic optic neuropathy (NAION) is presumably caused by circulatory insufficiency within the retrolaminar portion of the optic nerve head, which is supplied by the short posterior ciliary arteries. It is hypothesized that the apneic spells in OSAS patients might result in nocturnal hypoxemia, which could lead to acute or subacute optic nerve ischemia, mainly in patients with small optic discs [145, 146]. Approximately 70% of OSAS patients with NAION experience acute visual loss upon awakening [132, 147].

The presence and severity of diabetic retinopathy (DR), particularly proliferative DR and diabetic macular edema, have been found to be positively associated with severe OSAS. The formation of cottony exudates and increased retinal ischemic areas resulting from insufficient tissue oxygenation may be worsened by sleep apnea [144, 148].

Central serous chorioretinopathy (CSCR) can develop due to excessive stress or prolonged use of corticosteroids, but it can also be triggered by increased sympathetic activity due to elevated levels of endogenous catecholamines as a result of hypoxia caused by OSAS [144, 149].

Floppy eyelid syndrome (FES) is a particular eyelid disorder characterized by extensive lid laxity of the upper eyelid due to a decrease in elastin content, resulting in easy eyelid distortion and eversion with minimal traction. This eyelid condition is typically associated with chronic papillary conjunctivitis and DES. The prevalence of FES is greater in OSAS patients than in controls. The mechanisms leading to the development of FES in OSAS patients are likely related to subclinical systemic inflammation [150]. The correlation between FES, OSAS and obesity, defined by BMI > 29, has also been studied by Beis et al. They concluded that hyperelasticity of the lids was more significantly associated with obesity, but no association between FES and obesity was found [151]. These results were probably biased by the definition of obesity being limited to BMI and not including indices of visceral obesity.

In conclusion, OSAS is a very complex condition, with multiorgan complications, and treatment cannot be limited to a single symptom or feature of the disease. Rather, a multidisciplinary and integrated strategy is needed to achieve effective and lasting therapeutic effects [152].

## Conclusions

The prevalence of obesity, a chronic disease, is increasing, and obesity is now considered a global epidemic. Epidemiological studies have revealed an association between a high body mass index (BMI) and a wide range of chronic diseases that negatively affect people's health and quality of life and increase healthcare costs.

The incidence of eye diseases is also increasing worldwide, and these diseases have serious repercussions on quality of life as well as increasingly high costs for the community. There is now significant evidence that obesity is associated with several eye diseases, particularly cataracts, glaucoma, age-related macular degeneration, retinal vessel changes and occlusions, papilledema, and diabetic retinopathy; obesity also contributes to certain ocular diseases associated with obstructive sleep apnea (floppy eyelid syndrome, central serous chorioretinopathy, non-arteritic anterior ischemic optic neuropathy, and normotensive glaucoma), as well as refractive errors, amblyopia, and strabismus, which are often associated with monogenic syndromic obesity [9].

The ocular conditions underlying this association and the potential implications are very complex and seem to be related to body fat excess, mainly to the distribution of adipose tissue and its ectopic deposits. In fact, waist circumference (WC) and the waist/hip circumference ratio (WHR) have been observed to have stronger positive associations with eye diseases than BMI. The role of ectopic adiposity, mainly visceral-abdominal adiposity, and the beneficial effect of weight loss on ocular disorders are two crucial aspects of the relationship between obesity and eye diseases. However, further studies are needed to explore the impact of weight loss on preventing vision loss.

As the population ages, the prevalence of age-related eye diseases is expected to increase substantially worldwide. As a result, the number of people with moderate to severe visual impairment or blindness is expected to exceed 600 million patients by 2050 globally [5]. The same considerations also apply to excess weight, which is defined by the WHO as a real pandemic. We need to continue to address the upstream causes of obesity and the downstream effects on communities that are the most vulnerable to the obesity epidemic and have less access to new treatment options.

In the prevention and treatment of eye diseases related to obesity, lifestyle factors, especially diet and physical activity, as well as weight changes, both weight loss and weight gain, should not be overlooked. Obesity must therefore be considered a significant risk factor for eye diseases, hence the importance of a multidisciplinary and multidimensional approach to obesity, which also takes into account ocular health.

To support these conclusions and for public health policy purposes aimed at reducing the burden of ocular diseases due to obesity, we believe that ophthalmologists should not overlook body weight status when examining patients for specific eye diseases and, if necessary, refer the patient to a nutritionist. On the other hand, it could also be useful to consider the presence of eye problems during a general medical or dietary visit in the setting of a multidisciplinary approach for these patients.

## Level of evidence

Level V, narrative overview.

## What is already known on this subject?

Like obesity, eye diseases are known to be a significant public health problem. In particular, the prevalence of age-related eye diseases is expected to increase worldwide, especially as the population ages. Although the negative effects of obesity on health have been and are still the subject of multiple studies, the effects of excess weight on eye health are still poorly understood. In fact, obesity is a health problem that affects the whole body and has significant repercussions on the visual system. There are many eye diseases associated with obesity; among the most studied are glaucoma, cataracts, age-related macular degeneration (AMD), and diabetic retinopathy (DR).

## What does this study add?

The present review attempts to clarify the relationships of obesity and body fat distribution with multiple ocular pathologies. There is significant evidence that obesity is associated with several eye diseases, particularly cataracts, glaucoma, age-related macular degeneration and others, such as uveitis. Obesity also contributes to some ocular diseases associated with obstructive sleep apnea (OSA); the ocular conditions underlying this association appear to be related to excess body fat, mainly to the distribution of adipose tissue and its ectopic deposits. The role of adiposity distribution, mainly visceral-abdominal, and the beneficial effect of weight loss on ocular disorders are two crucial aspects of the relationship between obesity and ocular pathologies.

We trust in the usefulness of advising ophthalmologists not to neglect body weight and fat status when examining patients for specific eye diseases and, if necessary, to refer the patient to a nutritionist. On the other hand, it may also be useful to consider the presence of ocular problems during a general medical or dietary examination as part of a multidisciplinary approach for these patients.

## Data Availability

As per narrative review, no datasets were generated.
